# Genome editing: progress and challenges for medical applications

**DOI:** 10.1186/s13073-016-0378-9

**Published:** 2016-11-15

**Authors:** Dana Carroll

**Affiliations:** Department of Biochemistry, University of Utah School of Medicine, 15 N. Medical Drive East, Room 4100, Salt Lake City, UT 84112-5650 USA

## Abstract

The development of the CRISPR-Cas platform for genome editing has greatly simplified the process of making targeted genetic modifications. Applications of genome editing are expected to have a substantial impact on human therapies through the development of better animal models, new target discovery, and direct therapeutic intervention.

## Genome editing tools

Progress in biomedical research and its applications depends to a large extent on the methods available to investigate and manipulate cells and organisms. Until relatively recently, we had very limited capability to make intentional modifications to specific genes. This changed with the advent of programmable nucleases, which can induce very high levels of modification in arbitrarily selected genomic targets. First the zinc-finger nucleases (ZFNs), then transcription activator-like effector nucleases (TALENs), and most recently CRISPR-Cas nucleases (derived from clustered regularly interspaced short palindromic repeat (CRISPR) and CRISPR-associated (Cas) loci) have opened the door to making targeted genome alterations.

Remarkably, the essential CRISPR-Cas components were identified only a little over 4 years ago [1]. These consist of: 1) the Cas9 protein, which cuts DNA at a site determined by 2) a single-guide RNA (sgRNA) that carries a sequence (sometimes called the protospacer) that matches the DNA target, and (3) a short sequence in the target called the protospacer-adjacent motif (PAM) that is required for Cas9 binding. With experience from ZFNs and TALENs as a model, editing of genomic targets using CRISPR-Cas was quickly undertaken with resounding success [2]. The rapid adoption of CRISPR-Cas is due to several factors: unlike ZFNs and TALENs, only a single protein is required, and it does not have to be redesigned for each new target; target recognition is mediated simply by base pairing between the sgRNA and the target; production of new sgRNAs is very easy; and the system can be multiplexed by providing multiple sgRNAs. A number of variants of Cas9 are now available, each with potentially beneficial properties.

**Fig. 1:**
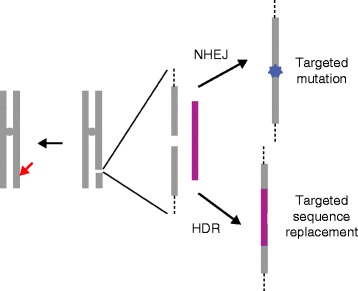
Illustration of the repair consequences of targeted DNA cleavage. The *red arrow* represents the targeted break at a single chromosomal site made by CRISPR-Cas (or zinc-finger nucleases (ZFNs) or transcription activator-like effector nucleases (TALENs)). The expanded view shows the break in the presence of a repair template (*magenta*). The break can be repaired by non-homologous end joining (*NHEJ*), leading to local insertions and deletions (indels) *(blue star*), or by homology-directed repair (*HDR*), incorporating sequences from the donor template. In most eukaryotic cells, NHEJ predominates, even in the presence of a homologous template. Because correction with donor sequences is often desired, considerable research is currently directed toward altering that balance

Like the earlier reagents, all the Cas9–sgRNA complex does is make a break in the genomic DNA target. The consequences of this break are the result of cellular DNA repair processes. Local, short insertions and deletions (indels) occur as a result of inaccurate non-homologous end joining (NHEJ). Sequence replacements can be introduced by homology-directed repair (HDR) with a DNA template supplied with the nuclease (Fig. 1). Both processes—local mutagenesis and sequence replacements—have obvious utility.

The CRISPR-Cas reagents have proven to be remarkably efficient in making genome modifications in a wide range of organisms and experimental settings. There has been concern, however, about specificity. While modifying the intended target, is Cas9 also introducing mutations at off-target sites? Changes in the sgRNA, in Cas9, and in delivery methods can enhance specificity [3], and these are highly relevant to human uses of the technology.

## Clinical uses: research

All research has the potential to have an impact on clinical practice, but here I focus on two broad categories: disease models and target identification. A key advance provided by genome editing has been the ability to extend models of human genetic diseases beyond mice and other common laboratory organisms. Disease-causing and predisposing mutations have been introduced into non-human primates and into large mammals with anatomic and physiologic characteristics more similar to humans. Among many examples, monkeys with muscular dystrophy were produced by injection of CRISPR-Cas materials directly into fertilized eggs [4]. Pigs with a predisposition to cardiac, neurological, and many other diseases have been created both by embryo injection and by somatic cell nuclear transfer [5]. These disease models facilitate the testing of various therapeutic approaches: pharmacological, nutritional, and genetic.

Broad screens with CRISPR-Cas are being used to identify new therapeutic targets. One example identified cellular genes required for infection by West Nile virus [6]. Other studies are directed toward defining pathways on which particular cancer cells uniquely depend for their growth. Discovery of novel targets will facilitate focused screens for novel therapies, including small molecule drugs.

## Clinical uses: somatic therapy

When a clear genetic contribution has been identified for any particular condition, genome editing of a patient’s own cells can be considered as a possible therapy. In fact, a clinical trial has been under way for several years for acquired immune deficiency syndrome (AIDS), using ZFNs to target the *CCR5* gene that encodes a co-receptor required by most strains of HIV-1 to infect T cells. Initial results showed efficient knockout of *CCR5*, persistence of modified cells, and absence of adverse effects [7].

This example illustrates the most accessible approach to somatic therapy by genome editing: manipulation of cells ex vivo and their return to the patient. For persistent benefit, the therapeutic modification should be made in long-term repopulating cells, such as hematopoietic stem cells. A variety of approaches is being pursued in the realm of hemoglobinopathies, including targeted correction of the sickle cell mutation and persistence of fetal hemoglobin [8]. As other stem cell approaches are mastered, including induced pluripotent stem cells from patients, genome editing can be applied to them as well. Prospects for in vivo genome editing are less rosy due to the challenges of delivering the materials effectively to the target tissues, but there is active research in this area.

Genome editing is being used to improve the performance of cell-based immunotherapies [9]. TALENs were used to produce ‘universal donor’ T cells, which were applied successfully to treat a young leukemia patient. Two recently approved clinical trials use CRISPR-Cas to inactivate the gene encoding programmed cell death protein PD-1 (which functions as an immune checkpoint and downregulates the immune system by preventing T-cell activation) to enhance the effectiveness of chimeric antigen receptor (CAR) T-cell therapies. In each of these cases, as with the *CCR5* trial, nucleases are being used to create targeted indels that inactivate a gene. Making targeted corrections by HDR is less efficient and more challenging, so advances in this area will be required.

## Clinical uses: germline modifications

Some genetic diseases do not readily lend themselves to somatic therapy. No effective gene therapy exists for cystic fibrosis, despite much effort, since the responsible gene was identified in the late 1980s. Cystic fibrosis affects multiple organs, and mortality usually results from defects in relatively inaccessible cells deep in the lung; thus, delivery of the therapeutic gene is very challenging.

In cases where somatic approaches do not look promising, researchers are considering making permanent changes to the genomes of human embryos. From a medical perspective, this has the advantage that gene correction will be permanent; neither the treated person nor any of his/her descendants will carry the disease allele. In principle, the procedure would involve manipulating embryos at a very early stage, in conjunction with in vitro fertilization. Two groups in China have published papers describing early steps in producing such modifications [10]. In these studies, and in other efforts just getting under way, there was no intent to create a pregnancy, and the embryos were never implanted.

The prospect of making heritable changes to the human genome has generated considerable concern. Do we know enough about human biology to predict confidently all the consequences of such modifications? Are the procedures safe enough, or will they induce unwanted mutations and consequences? Will the technology be used to attempt “enhancements”—greater intelligence, athletic prowess, attractive physical features—rather than disease treatments?

## Concluding remarks

My view is that genome editing technology is simple enough that it will certainly be used for reproductive editing in the foreseeable future. It should not be done today because the methods are neither efficient nor safe enough to ensure beneficial outcomes. Nonetheless, there will be interest from patients and advocacy groups in employing technology that can help prevent debilitating diseases. The role of research should be to make improvements that will ensure safety and efficacy. This will require research with human embryos, under very stringent criteria for approval and monitoring. Key issues will be, first, minimizing off-target effects. Considerable progress has been made in this area, but the issue will have to be assessed specifically for each new target. Second, it will be important to improve the efficiency of targeted correction by HDR, perhaps by inhibition of the NHEJ pathway. And last, researchers need to develop effective delivery methods for in vivo treatments with CRISPR-Cas. Viral vectors show promise, but the targeting and efficiency of these vectors can still be improved.

Equally important is careful consideration of the societal and ethical issues raised by both somatic and reproductive genome editing. Beyond assessing our comfort with gene manipulation technology, we must also consider what genetic conditions are legitimate candidates for modification and how the benefits of any therapy will be distributed to the people who need it most. As an example of the former issue, is hereditary deafness a condition that should be eliminated? Most deaf people are very high-functioning, and many would not identify their situation as needing to be fixed. Sickle cell disease falls into the latter category. A good deal of research is directed at modifying patient stem cells, but the approaches all involve laboratory manipulations that are time-consuming, expensive, and not readily transported to regions of the world where the disease is endemic. How can a genetic therapy be made widely available?

International discussions and assessments of genome editing and its implications are under way, but are still at early stages. The prospects for beneficial medical uses of genome editing are bright, and research is being pursued very broadly. How these benefits are ultimately employed will depend on efforts both inside and outside the laboratory and the clinic.

## Abbreviations

CAR: Chimeric antigen receptor
Cas: CRISPR-associated
CRISPR: Clustered regularly interspaced short palindromic repeats
HDR: Homology-directed repair
indels: Insertions and deletions
NHEJ: Non-homologous end joining
PAM: Protospacer-adjacent motif
sgRNA: Single-guide RNA
TALENs: Transcription activator-like effector nucleases
ZFNs: Zinc-finger nucleases
